# Complete genome sequence of *Thermincola carboxydiphila* DSM 17129^T^ isolated from a hot spring of the Lake Baikal area

**DOI:** 10.1128/mra.00822-26

**Published:** 2026-09-17

**Authors:** Florian P. Rosenbaum, Tim Böer, Volker Müller, Rolf Daniel, Anja Poehlein

**Affiliations:** 1 Department of Molecular Microbiology & Bioenergetics, Institute of Molecular Biosciences, Johann Wolfgang Goethe University 9173, Frankfurt, Germany; 2 Genomic and Applied Microbiology & Göttingen Genomics Laboratory, Institute of Microbiology and Genetics, Georg-August University Göttingen 9375, Göttingen, Germany; University of Rhode Island, Kingston, Rhode Island, USA

**Keywords:** *Thermincola*, *carboxydiphila*, carboxydotrophic, hydrogenogenic, acetogen, thermophilic

## Abstract

We report on the complete genome sequence of the carboxydotrophic hydrogenogenic, thermophilic, and alkalitolerant acetogen *Thermincola carboxydiphila* (DSM 17129^T^). The genome harbors a circular chromosome with a size of 3.519 Mbp and a GC content of 45.6%. The genome contains 3,490 genes in total.

## ANNOUNCEMENT

*Thermincola carboxydiphila* (DSM 17129ᵀ) is an anaerobic, thermophilic, and alkalitolerant bacterium that was first isolated and described by Sokolova et al. in 2005 (1). *T. carboxydiphila* is an obligate carboxydotrophic bacterium performing the water–gas shift reaction during growth on CO. This raises interesting physiological and biochemical questions, such as how physiological electron carriers are reduced or how is biomass synthesized under these conditions?

Despite its significance, a high-quality complete genome sequence was not available. To allow detailed genome-based studies, the strain was chosen for sequencing (2, 3). We report the complete genome of the type strain *T. carboxydiphila* DSM 17129^T^, which was originally isolated from a hot spring of the Lake Baikal Region, Russia, by Sokolova et al. (1). Cultures were inoculated from an actively growing culture obtained from the German Collection of Microorganisms and Cell Cultures (DSMZ, Braunschweig, Germany) and cultivated in 20 mL of DSM 1028 medium at 55°C under anaerobic conditions. Cells were harvested by centrifugation at 10,000 × *g* for 10 min. Genomic DNA was extracted using the NucleoMag DNA Bacteria Kit according to the manufacturer’s instructions (Macherey-Nagel, Düren, Germany).

The Nextera XT DNA sample preparation kit was used to prepare sequencing libraries, which were sequenced using a MiSeq system and v3 chemistry (2 × 300 bp, 600 cycles) following the instructions of the manufacturer (Illumina, San Diego, CA, USA). Nanopore sequencing libraries were prepared with 600 ng high-molecular weight DNA without prior size selection using the Native Barcoding Kit 24 V14 (SQK-NBD114.24; barcode 2) as recommended by the manufacturer (Oxford Nanopore Technologies, Oxford, UK). Nanopore sequencing was conducted for 89 h using the MinION device Mk1B, a SpotON flow cell R10.4.1, and the MinKNOW software v25.05.12, as recommended by the manufacturer (Oxford Nanopore Technologies). Default parameters were used for all software unless otherwise specified. Demultiplexing and base calling of Nanopore reads were performed with the Dorado v1.2.0 software in SUP mode (Oxford Nanopore Technologies).

Nanopore reads were quality-filtered with Fastp v0.4.1 (4, 5) and subsampled with Trycycler v0.5.6 (6) for the generation of *de novo* genome assemblies using Flye v2.9.6 (7) and Canu v2.3 (8). Flye assemblies were processed by trimming overlaps and performing circularization using the assembler’s integrated module. For Canu assemblies, overlaps were trimmed using the canu_trim.py script (Trycycler repository: https://github.com/rrwick/Trycycler/blob/main/scripts/canu_trim.py), followed by circularization employing the reconcile module of Trycycler. Assemblies were combined using Trycycler consensus and polished with the full Nanopore data with Medaka v2.1.1 using the model “*r1041_e82_400bps_bacterial_methylation*.” Illumina reads were quality-filtered with Fastp v1.0.1 (4, 5) and subsequently aligned to the consensus sequence with the Burrows-Wheeler Aligner v0.7.19 (9). Polypolish v0.6.1 (10) and Pypolca v0.4.0 (11) were used for polishing. The genome sequence was reorientated using Dnaapler v1.3.0 (12) and annotated with the National Center for Biotechnology Information (NCBI) Prokaryotic Genome Annotation Pipeline (PGAP) v6.11 (13). Genome quality assessments were performed with CheckM2 v1.1.0 (14) and BUSCO v6.0.0 (15). Sequencing statistics of Nanopore reads were determined using Minimap2 v2.30 (16), Samtools v1.22.1 (17), and Cramino v1.1.0 (18) and statistics of Illumina reads using Bowtie2 v2.5.4 (19), Samtools (17), and Qualimap2 v2.3 (20). Detailed genome statistics of *T. carboxydiphila* are summarized in Table 1.

**TABLE 1:** Sequence statistics and genome features of *T. carboxydiphila* DSM 17129^T^ based on PGAP annotation and CheckM analysis (13, 14)

Features	*T. carboxydiphila* DSM 17129^T^
Chromosome size (bp)	3,518,842
Number of Illumina reads raw (300 bp)/trimmed (avg. 267 bp)	5,112,310/4,728,122
Number of Nanopore raw/trimmed reads/mean length (bp)/N50 (bp)	955,604/611,320/1,602/3,295
Chromosome mean coverage	358-Fold Illumina/526-fold Nanopore
GC content (%)	45.6
Genes (total)	3,490
Coding density (%)	86.2
CDS (total)	3,425
CDS with predicted function	2,915
CDS without predicted function	510
rRNAs (5S, 16S, 23S)	3, 3, 3
tRNAs	52
tmRNAs	1
ncRNAs	3
Pseudogenes	39
Riboswitches	43
CRISPR arrays	5
CheckM2 completeness score (%)	99.93
CheckM2 contamination score (%)	0.55
BUSCO (lineage: bacteria_odb12)	C: 99.1% [S: 99.1%, D: 0.0%], F: 0.0%, M0.9%, n: 116
BUSCO (lineage: eubacteriales_odb12)	C: 97.5% [S: 97.1%, D :0.4%], F: 0.4%, M: 2.1%, n: 240

## Data Availability

The genome sequence and annotation were deposited with the NCBI genome accession JBWXHF000000000 under BioProject PRJNA1435825 and BioSample SAMN56455830. Raw read data were deposited in the NCBI Sequence Read Archive (SRA) under accession numbers SRR38013484 (Illumina) and SRR39869403 (Nanopore).
